# Wavelet-based identification of DNA focal genomic aberrations from single nucleotide polymorphism arrays

**DOI:** 10.1186/1471-2105-12-146

**Published:** 2011-05-11

**Authors:** Youngmi Hur, Hyunju Lee

**Affiliations:** 1 Dept of Applied Mathematics and Statistics, Johns Hopkins University, Baltimore, MD, USA; 2 Dept of Information and Communications, Gwangju Institute of Science and Technology, Gwangju, South Korea

## Abstract

**Background:**

Copy number aberrations (CNAs) are an important molecular signature in cancer initiation, development, and progression. However, these aberrations span a wide range of chromosomes, making it hard to distinguish cancer related genes from other genes that are not closely related to cancer but are located in broadly aberrant regions. With the current availability of high-resolution data sets such as single nucleotide polymorphism (SNP) microarrays, it has become an important issue to develop a computational method to detect driving genes related to cancer development located in the focal regions of CNAs.

**Results:**

In this study, we introduce a novel method referred to as the wavelet-based identification of focal genomic aberrations (WIFA). The use of the wavelet analysis, because it is a multi-resolution approach, makes it possible to effectively identify focal genomic aberrations in broadly aberrant regions. The proposed method integrates multiple cancer samples so that it enables the detection of the consistent aberrations across multiple samples. We then apply this method to glioblastoma multiforme and lung cancer data sets from the SNP microarray platform. Through this process, we confirm the ability to detect previously known cancer related genes from both cancer types with high accuracy. Also, the application of this approach to a lung cancer data set identifies focal amplification regions that contain known oncogenes, though these regions are not reported using a recent CNAs detecting algorithm GISTIC: SMAD7 (chr18q21.1) and FGF10 (chr5p12).

**Conclusions:**

Our results suggest that WIFA can be used to reveal cancer related genes in various cancer data sets.

## Background

With the recent advances of cancer studies at a molecular level, DNA copy number aberrations (CNAs) have been studied as important causes and consequences in the initiation, development, and progression of cancer. To date, many researchers have focused on the detection of chromosomal regions having amplifications and deletions using arrays of comparative genomic hybridization (CGH) data sets. These studies have generated valuable observations about cancer metastasis [1-7]. For example, it is now known that many oncogenes and tumor suppressor genes are located in regions of amplifications and deletions, and that chromosome regions with aberrations can be used to distinguish between cancer types. Also, new cancer related genes have been discovered. These advances have been accelerated by the development of computational methods and software [8-14]; segmentation and denoising methods such as circular binary segmentation (CBS) [8], wavelets [9], and the Gaussian-based likelihood approach (GLAD) [10] have been developed in order to identify true aberrations from background noise in a single sample. And with the accumulation of copy number aberration data sets, it has become increasingly important to find concordant aberrations in multiple samples. Thus, algorithms such as the minimum common region (MCRs) [15] and significance testing for aberrant copy number (STAC) [16] have been developed to address this issue.

However, even though each method can identify aberrant regions, these regions are not concordant between the different methods. As one possible explanation for this lack of concordance, Beroukhim et al. (2007) [17] assumed that many aberrations randomly occur, though most methods do not explicitly consider the background rate of random aberrations. For instance, most locations of chr7 and chr10 are amplified and deleted, respectively, in short-term survival patients of glioblastoma multiforme (GBM) [18], though only a few of their genes are known oncogenes and tumor suppressors in GBM. As such, if random aberrations are not considered, most chr7 and chr10 genes will be regarded as relevant. Hence, an important issue is to distinguish cancer driving genes, i.e., genes involved in cancer development, from broad chromosomal aberrations. Fortunately, the amount of aberrations of driving genes has been observed to be larger than in their neighboring genes, and these aberrations are likely to occur consistently across multiple cancer patients. A few algorithms, such as the genomic identification of significant targets in cancer (GISTIC) [17], have been developed in attempts to incorporate these issues and are used to detect focal aberrations. Note that the term "focal aberrations" is used here to refer to relatively short, but consistently aberrant, regions in multiple samples. The use of GISTIC revealed that these focal aberrations contain many cancer related genes. In a comparison of GISTIC to MCR [15], via three independent data sets, GISTIC consistently identified more cancer related genes than MCR. In GISTIC, it first selects copy number aberration regions by applying a segmentation method to each sample, and then sums the amount of aberrations from the multiple samples. Then, differences between the aberrations and their neighbors are computed using a peel-off method. However, GISTIC has an inherent weakness: differences between neighbors in individual samples may cancel out since it summates log2 ratios in all aberrant samples first. The important difference between GISTIC and our proposed approach is that we first consider the differences between neighbors in an individual sample, before identifying focal regions in multiple samples. In this study, we propose a novel algorithm, referred to as the wavelet-based identification of focal genomic aberrations (WIFA), to address the following issues: (i) distinguish signals from noise among probes having high aberrations, (ii) detect focal aberrations by considering the differences between aberrations and their neighbors, as well as the amount of aberrations, and (iii) consider the consistency of aberrations in multiple samples.

Wavelet analysis is a mathematical technique for representing data. Wavelets can be used to remove noise from observed data (contaminated by noise) while preserving important features of true data; this process is called wavelet denoising. In this study, we use a variant of the translation-invariant level dependent wavelet denoising method in [19] to obtain translation-invariant approximations of the smooth (low-frequency) part of true data *y_LOW_*, and of the local (high-frequency) behavior of true data *y_HIGH_*, from the observed data *y*. In brief, *y_LOW _*is based on the averages of the neighboring values of *y*, and *y_HIGH _*is based on the differences of neighboring values of *y*, followed by thresholding. Thresholding is only performed in *y_HIGH _*since it is likely that noise would be more pronounced in the high-frequency content. After obtaining *y_HIGH _*via the wavelet analysis, we obtain  for each sample by adjusting some obvious artifacts in *y_HIGH_*, and then cluster continuous focal aberrations across multiple samples. By applying this approach to GBM and lung cancer data sets, we are able to find previously known cancer related genes in the focal aberrations. In addition, a similar procedure based on *y_LOW _*enables us to detect broad regions of chromosomal aberrations.

The difficulty of assessing the performance in detecting focal aberrations is that the true answer is often not known, since regions containing cancer related genes still need to be revealed. Hence, we compare genes identified by our approach to known cancer genes obtained from GISTIC [17]. Based on this comparison, in addition to confirming regions identified by GISTIC, we are able to find new regions not previously identified by GISTIC; literature shows that these new regions contain known oncogenes. In addition, WIFA is compared to STAC and MCR, outperforming these two methods both in the simulation and GBM data. The source code for WIFA is available at http://www.gcancer.org/wifa/WIFA.html.

## Materials and methods

### Materials

We collected and reanalyzed three single nucleotide polymorphism (SNP) data sets: 154 GBM tumor samples [17], 178 GBM tumor samples [20], and 371 lung tumor samples [21]. We downloaded the signal intensities of the data sets from either the websites of the original publications or the GEO database. We used all chromosomes except X and Y. Both GBM data sets were generated from an Affymetrix 100K SNP microarray, and the lung cancer data set was from 250K Sty SNP arrays. Since the 100K SNP array consisted of independent 50K Xba and 50K Hind arrays, we then merged these two arrays along the chromosome positions. Next, to calculate copy number changes from signal intensities, we applied the following procedure (similar to original publications): (i) signal intensities were transformed using the log2 transform to make the noise constant; (ii) for each sample, the median value across all probes was subtracted from the probes; (iii) to obtain the log2 ratio for tumor samples compared to the normal samples, log2 transformed normal samples were subtracted from the log2 transformed tumor samples; and (iv) to remove copy number variants (CNVs) that occur in normal population, positions with CNVs obtained from [22] were omitted from the data sets.

### WIFA methodology

**Figure 1:**
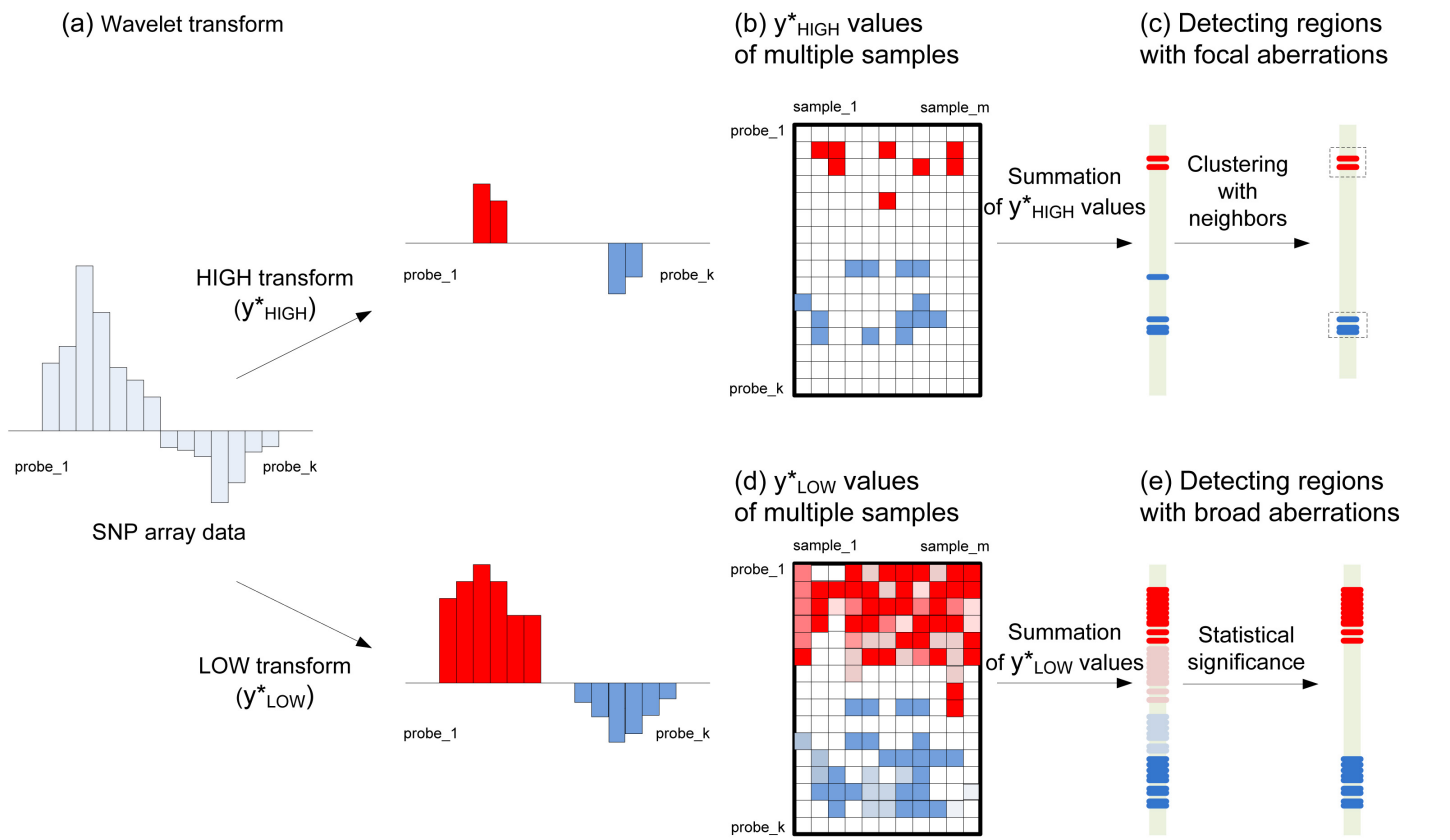
**Schematic of our approach**. (a) From a SNP array sample, , representing differences of aberrations with neighbors, and  values, showing the overall aberrations, are generated using the wavelet transform. (b) The  from multiple samples are summed in order to combine aberrations from multiple samples. (c) Focal aberrations are identified by clustering probes with their neighbors. (d) The  values from multiple samples are summed to identify aberrations from multiple samples. (e) Broad aberration regions are identified if the summation of  values across multiple samples are larger than a threshold of statistical significance the size of a chromosome arm.

Figure 1 illustrates the WIFA method for detecting broad and focal aberrations in SNP array data sets using the wavelet transform. The procedure for detecting focal aberrations is as follows. First, the difference between the aberrations and their neighbors is measured for the SNP array data using the wavelet transform and a post-processing step; this process is called a HIGH transform and generates . Probes that have a larger difference with their neighbors than a given threshold value are likely to have nonzero values in . Thus,  can be used to distinguish driving aberrations from passenger aberrations because the amount of aberrations for driving genes is usually larger than for their neighbors. Second, the significance of probes for indicating the driving gene in multiple samples is calculated as the sum of the values in  from multiple samples, and is referred to as . This value is generated based on the assumption that both the number of samples with aberrations and the amount of aberrations are important indicators for discovering driving aberrations. Third, probes having nonzero  values are clustered with neighbors having nonzero  values within a certain distance. Since chromosome positions are also important for identifying candidate cancer-related genes, we consider sets of neighboring probes in this third step instead of using the information from individual probes. To prioritize these clusters, the score of each cluster is determined by summing the probes' values in  in the cluster. Similar to the procedure for detecting focal aberrations, broad aberrations are identified using ;  values are calculated for each sample and then summed for all samples, denoted as . The statistical significance of the aberrations is subsequently calculated as shown in Figure 1(d)-(e).

### Wavelet transform and its use in WIFA

#### Wavelet transform

Let *J *and *L *be integers such that 1 ≤ *L *≤ *J *- 1. The (discrete) wavelet transform (WT) maps a given data set *y *of length 2^*J *^into the scaling coefficients *s *:= {*s*_*L*,*t*_: *t *= 0, 1, ..., 2*^L ^*- 1} and the wavelet coefficients *w *:= {*w*_*j*,*t*_: *j *= *L*, *L *+ 1, ..., *J *- 1; *t *= 0, 1, ..., 2^*j *^- 1}. Note that WT is linear and can be represented by a 2^*J *^× 2^*J *^orthogonal matrix *W*. WT depends on the specific wavelet selected. In this paper, we use a WT based on the Haar wavelet. The Haar wavelet transform is used to simply pair up input values, storing the difference and passing the average, and it repeats this process recursively, pairing up the averages to provide the next level--finally resulting in 2*^L ^*averages (stored in the scaling coefficients s) and 2^*J *^- 2*^L ^*differences (that are stored in the wavelet coefficients *w*). For more details about wavelets, refer to [23], [24], and [25].

#### Wavelet procedure for WIFA

A drawback of the traditional WT is that it is not translation-invariant. In attempts to remedy this problem, a number of translation-invariant wavelet transforms have been employed [9,19,26]. Among the available translation-invariant wavelet transforms, we use the stationary wavelet transform by [27] for our WIFA methodology.

Suppose that a single tumor sample is fixed, and that a chromosome of the sample has *n *= 2^*J *^locations for some positive integer *J*. We then denote *y_i _*as the observed copy number change at the *i*-th genomic location *x_i _*for *i *= 1, ..., *n*. We assume that the genomic locations *x_i _*are fixed, known and equally spaced; for a wavelet analysis with unequally spaced data, see example [28]. We further assume that for *i *= 1, ..., *n*, the observed copy number change can be expressed as(1)

where *f *is an underlying function representing the true copy number change, and *ε_i _*is a stationary Gaussian noise with a zero mean value [19].

The basic principle of wavelet denoising then becomes to identify and zero out the wavelet coefficients *y *:= {*y_i _*: *i *= 1, ..., *n*} that are likely to contain noise, and to estimate {*f *(*x_i_*): *i *= 1, ..., *n*} in (1). Instead of the usual wavelet denoising procedure [29], we use the following modified steps as the main wavelet denoising procedure for our methodology:

(Step 1) Given the data *y *of length *n *= 2*^J^*, and an integer *L *such that 1 ≤ *L *≤ *J *- 1, compute *W y *= {*s*, *w*}. For an integer *M *≥ *L*, let *w_MID _*be the wavelet coefficients *w *of *y *with levels *j *= *L*, *L *+ 1, ..., *M *- 1, and *w_HIGH _*be the wavelet coefficients *w *of *y *with levels *j *= *M*, *M *+ 1, ..., *J *- 1.

(Step 2) Define *T_LOW_*:{*s*, *w_MID_*, *w_HIGH_*} ↦ {*s*, 0, 0} and , where  is obtained from *w_HIGH _*by thresholding using a hard threshold function [30] with the threshold value  for each level *j *= *M*, *M *+ 1, ..., *J *- 1. Here,  is the estimate of the noise variance for the wavelet coefficients at level *j*, *n_j _*is the length of the subsignal at level *j*, and *C *is a constant to be determined later.

(Step 3) Let *S *represent a shift operator of the one time unit [27]. Then, compute the translation-invariant low-frequency and high-frequency approximations *y_LOW _*and *y_HIGH _*defined as

The threshold value  used in (Step 2) is a variant of the threshold value used in [19]. After (Step 1)-(Step 3), we obtain *y_LOW _*and *y_HIGH_*. Note that *y_LOW _*gives a translation-invariant approximation of the smooth (low-frequency) part of the true data, which provides rough estimate for detecting a broad region of chromosomal aberrations. This value is based on the Haar scaling coefficients, which can be considered as averages of neighboring values of *y*. On the other hand, *y_HIGH _*gives a translation-invariant approximation of the local (high-frequency) behavior of the true data, which provides a rough idea for detecting the focal aberration of chromosomes; *y_HIGH _*is based on Haar wavelet coefficients -- which are differences between the neighboring values of *y*--and the threshold.

Dividing the wavelet coefficients depending on the level has been used in many studies (see [19] and [31]), although the exact form may vary. The main difference between our method and other level-dependent wavelet denoising methods is that we concentrate only on the low-frequency scaling and high-frequency wavelet coefficients, and do not consider the mid-frequency wavelet coefficients. To do this, we add the parameter *M *to the usual wavelet thresholding process; from the discussion in the Results section, this parameter allows us to identify focal genomic aberrations more effectively.

The values of *y_LOW _*for all chromosomes of a given sample *y *are obtained simply by processing each chromosome separately, and then concatenating the values of *y_LOW _*for each chromosome; similarly, the values of *y_HIGH _*for all chromosomes can be found.

Next, let us explain how we treat the problem of the boundary of each chromosome. In brief, the problem of the boundary is caused by our previous assumption that the chromosome has *n *= 2^*J *^locations for a positive integer *J*, which may not hold true in general; for a more detailed discussion about boundary conditions, refer to [32]. We handle this boundary problem by extending each chromosome first symmetrically and then periodically. Our experiments show the effectiveness of this method. Other parameters used in our methodology include:

• Constant *C *in the threshold value : in (Step 2), we use the threshold value  to threshold the high-frequency wavelet coefficients at level *j*. A smaller *C *would allow more nonzero values in *y_HIGH_*.

• Level *L*: parameter *L *can be as small as 1 and as large as *J *- 1. A smaller *L *would increase the coarseness of the *y_LOW _*approximation, whereas a larger *L *would make it finer.

• Level *M *: parameter *M *can be as small as *L *and as large as needed. A smaller *M *would produce a *y_HIGH _*with more nonzero values, whereas a larger *M *would produce a *y_HIGH _*with fewer nonzero values. Since this is not a standard parameter in wavelet literature, we pay special attention to it and discuss its effect on our methodology by varying *M*. See the Results section.

In the Results section, we further discuss which values of the above parameters *C*, *L*, and *M *are used for each of the data sets in our experiments. To implement the wavelet transforms, we used WaveLab http://www-stat.stanford.edu/%7Ewavelab/Wavelab_850/index_wavelab850.html.

### Identification of broad and focal aberrations in WIFA

#### Identification of focal aberrations

**Figure 2:**
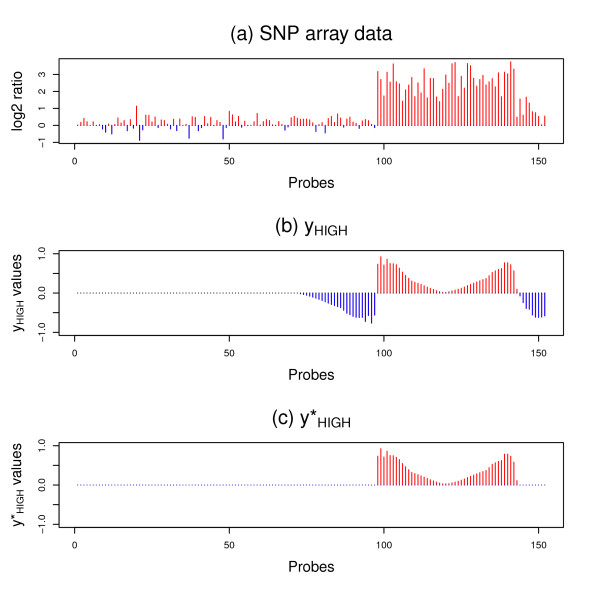
**Processing *y_HIGH _*values**. (a) log2 ratios of 152 probes of SNP array data are shown from one sample. (b) With the wavelet transform, *y_HIGH _*values representing neighboring values of the denoised sample are generated. Note that negative values are generated because positions next to amplifications also show large differences with their neighbors. (c) In this example, negative *y_HIGH _*values are set to zero to generate  because they do not represent true aberrations. To keep only *y_HIGH _*positions with amplifications, we pick a position with the highest absolute log2 ratio value among the consecutive positions with nonzero *y_HIGH _*values, and then assign zero to the positions having a different sign in the *y_HIGH _*values.

The values of *y_HIGH _*generated from the wavelet transform indicate the difference between the neighboring values of the denoised sample. To use this information for focal aberration detection in the WIFA method, we process the *y_HIGH _*values. In Figure 2(a), we draw the log2 ratio of 152 probes for a single sample, a part of the GBM SNP array data [17], in which there are strong amplifications between the 98th and 143th probes. The *y_HIGH _*values obtained by the wavelet transform are then presented in Figure 2(b). Note that negative *y_HIGH _*values are generated around the positive *y_HIGH _*values of the 98th and 143th probes. This negativity is due to the fact that the positions next to amplifications also have large differences with their neighbors. To keep only *y_HIGH _*positions with amplifications, we select the position with the highest absolute log2 ratio value among the consecutive positions with nonzero *y_HIGH _*values, and then assign zero to the positions with a different sign in the *y_HIGH _*values. This process generates , as shown in Figure 2(c). A similar process is then performed on the deletions.

**Figure 3:**
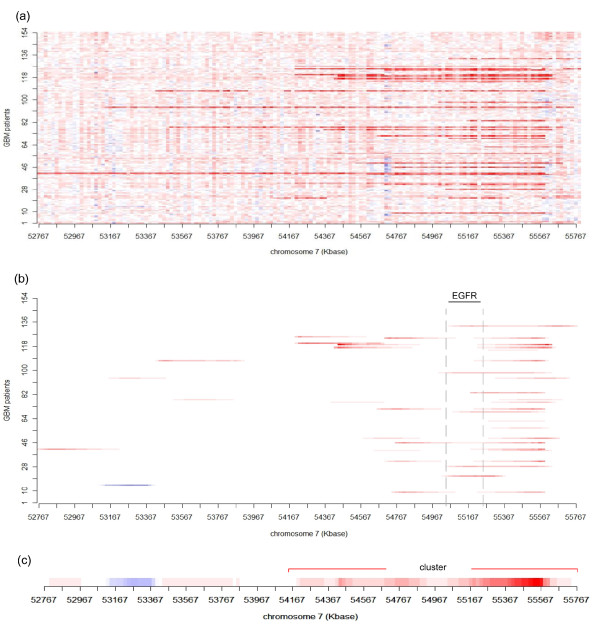
**Comparing log2 ratio of SNP array data with  for the GBM data set**. (a) log2 ratios of the SNP array data for 154 GBM patients [17] are drawn for the region from 52,767 to 55,790 KB in chr7. Stronger red presents a larger log2 ratio, representing amplifications. Amplifications are abundant in this region. (b)  in the corresponding regions of (a) are presented. Probes with nonzero values in  are drawn in red (blue) for amplifications (deletions). EGFR, a GBM gene, is located in this region. (c)  from multiple samples are drawn for the same region. A part of the region from 54,145 to 55,790 KB is detected as a focal aberration.

After obtaining , WIFA considers the two following issues. Let  be the value of  at a position *p*. First, if for a given probe *p*, , and for its consecutive probes *k *on both sides, , the *p *might represent CNVs that are abundant in a normal population, instead of CNAs. Thus, we set . Second, we attempt to determine focal aberrations that are consistent across multiple samples. Figure 3(a) shows the log2 ratio of the SNP array data of 154 samples in the same region as in Figure 2(a); this region consists of 152 probes. The sample used in Figure 2 is one of these samples, and red (or blue) indicates the amplifications (or deletions, respectively). The HIGH transformed values of Figure 3(a) are then drawn in Figure 3(b). As shown in both Figures 2(c) and 3(b), a HIGH transform value assigns relatively big nonzero values to the boundaries of aberrations and relatively small (or zero) values to the middle of aberrations. Note, therefore, that zero  values do not always indicate that there are no aberrations. Hence, consecutively or nearly consecutively occurring nonzero  values across multiple samples might reflect the presence of true focal aberrations. As such, we sum the  of the multiple samples shown in Figure 3(c), subsequently represented as .

In order to identify the focal aberration regions, we consider the neighboring positions together instead of as a single position. For this task, we consider groups of positions having positive (or negative)  values located within 1 MB along the chromosome. Then, in order to find regions of focal aberrations in a group, we construct clusters such that the two closest positions in a cluster having positive (or negative)  values are located within a distance *d*. From the clusters in a group, we select a cluster *c *having the maximum score . In the clustering process, clusters containing nonzero  values from only a single patient are removed. Then, statistically significant clusters are ranked based on their *S*(*c*) scores. In Figure 3(c), the cluster on the right is a focal aberration that contains a known cancer related gene (EGFR).

A statistical significance of each cluster is calculated based on the null hypothesis that consecutive aberrations in each sample are independent from those in other samples. Here, the number of permutations *N *and the significant value *α *are used to estimate the significance of clusters.

1. In each sample, segments of aberrations (a set of consecutive probes with ) are randomly positioned on a chromosome. This random positioning is then applied to all samples, generating randomly permuted data from the multiple samples. This permutation approach is described in detail in [11].

2. The process for detecting focal aberrations in multiple samples is subsequently applied to the randomly permuted data, generating a set of clusters.

3. Steps 1 and 2 are repeated *N *times. Let the max score of clusters from the *i*th permutation be the *max_score*(*i*).

4. The *p*-value of each cluster *c *of the observed data *P*_cluster_(*c*) can be calculated by comparing scores from the permuted and observed data:(2)

where *S*(*c*) is the score of a given cluster *c*, and *I *denotes the indicator function.

5. Clusters with P_cluster_(c) less than *α *are considered statistically significant.

In this paper, we use *N *= 1,000 and *α *= 0.1, and the permutation and calculation of *P*_cluster _is performed for each chromosome.

#### Identification of broad aberrations

After *y_LOW _*values are generated from the wavelet transform for each sample, we use *y_LOW _*as ; *y_LOW _*values do not require a processing step, contrary to *y_HIGH_*. To integrate multiple samples, we sum  values from multiple samples, referred to as . Note that if all probes in a chromosome arm are statistically significant in , we consider it a broad aberration. We then calculate the statistical significance of  in the following way. The null hypothesis for  is that  is independent among samples, so the summation of , , is the same across all probes in the chromosomes. To generate the null distribution, we first construct a histogram *h_i _*of  in a single sample *i *by splitting  values into bins at intervals of 0.01. Next, the distribution of  is calculated by the convolution of *h_i _*of all samples, and the *p*-value of the observed  is calculated by summing the probabilities from the tail of the null distribution to the observed  value. The *p*-value is separately calculated for amplifications and deletions. For the correction of multiple tests, *p*-values are converted into *q*-values [33]; the *p*-values of  are similarly calculated. This approach is similar to the calculation of statistical significance of aberrations used in [17]. Note that the *p*-values of  are calculated for each probe, and the *P*_cluster _discussed above is calculated for each cluster.

## Results

### Broad and focal aberrations in glioblastoma

**Figure 4:**
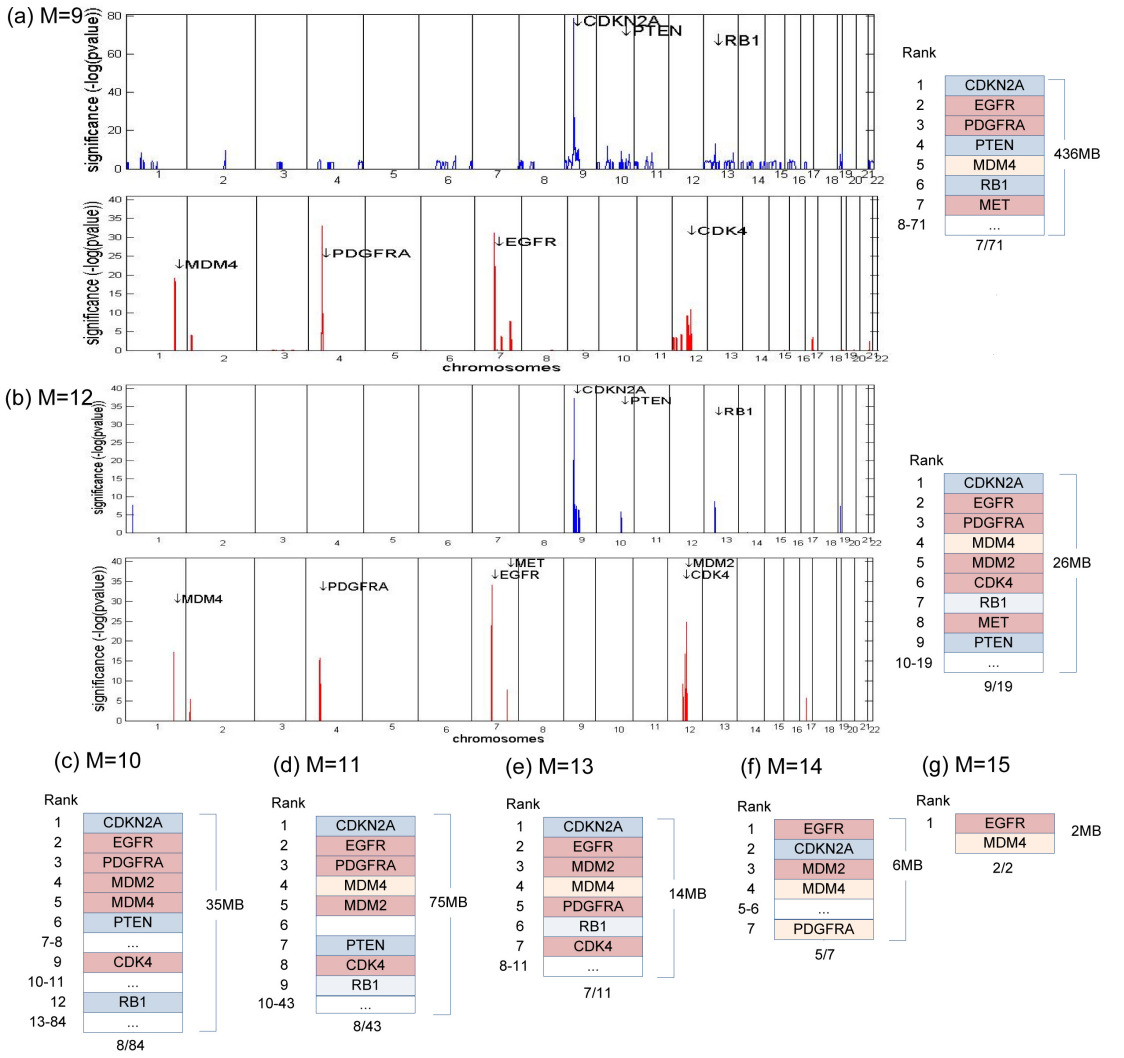
**Focal aberrations in GBM data with several *M *values**. Focal aberrations of GBM data [17] are shown.  and its corresponding *p*-values are generated using *M *values from 9 to 15. (a)-(b) On the left, *p*-values of  are drawn across chromosomes with GBM related genes for *M *values of 9 and 12. Deletions (amplifications) are represented in blue (red). On the right, statistically significant clusters with GBM related gene symbols are shown. For example, in (a), 71 clusters are generated and the top seven clusters include seven GBM genes. These clusters span 436 MB chromosomal regions in total. Clusters with deletions (amplifications) are represented in blue (red). As *M *increases, the number of clusters decreases. Although the values of *M *change, GBM genes are still located in the highly ranked clusters. When *M *= 12, a cluster containing RB1 (MDM4) is indicated in light blue (light red), which denotes that RB1 (MDM4) lies slightly out of the cluster. (c)-(g) Clusters with *M *values of 10, 11, 13, 14, and 15 are shown for GBM genes.

We applied our approach to 154 GBM tumor samples [17]. After testing different values of *C*, *L*, and *M *(cf. Methods section), we selected *C *= 1.94, *L *= 9, and *M *= 12. Our experimental results for different values of M are shown below (experiments for different values of *C *and *L *are not shown). Since *J_total _*:= ⌈log 2(*n_total_*)⌉ = 17, where *n_total _*is the number of total probes in the data set, *L *= 9 for this data set roughly indicates that we average the log2 ratios of  probes in the 100K SNP array to obtain *y_LOW_*. In order to identify broad aberrations, we apply the LOW transform and calculate the *q*-values of . Here, amplifications and deletions in the size of a chromosome arm are considered broad aberrations, with a threshold *q*-value of 0.01. As shown in Additional File 1(a) and 1(b), chr7, 8q, 17q, 19p, and 20 are amplified, and chr6q, 9p, 10, 13, 14, and 22 are deleted in the size of a chromosome arm. Next, using the HIGH transform, our model is able to detect clusters with focal aberrations; we used *d *= 100 KB to construct clusters. Since EGFR, MDM2, PDGFRA, MDM4, CDK4, MET, CDKN2A, PTEN, RB1, CDK6, and MYC have been reported as important GBM related genes [34], we investigate whether the clusters contain these genes. In addition, we investigate the effect of different *M *values for detecting focal aberrations. We use *M *values ≥ 9 since *M *can have values from *L*, as described in the Methods section. The number of probes with nonzero values in  is the largest when *M *= 9; Figure 4(a) shows that 71 statistically significant clusters are generated for this value. In these clusters, seven GBM related genes are ranked as the top seven clusters. As *M *increases, the number of clusters and the number of nonzero probes in  decreases. Indeed, in Figure 4(g) only two clusters are generated when *M *= 15. In the range *M *= 10-12, eight or nine GBM genes are located in the highly ranked clusters; this observation shows that our method highly ranks clusters containing important GBM genes regardless of the *M *value. However, if we consider the size of the chromosomal region containing the identified focal aberrations, there is a preferred choice for *M *value. When *M *= 9, among all 71 clusters spanning 436 MB, the top seven clusters contained seven GBM genes. On the other hand, when *M *= 12, 19 clusters spanning 26 MB included nine GBM genes in the top nine clusters. Hence, we use *M *= 12 for further analysis. As shown in Figure 4(b), these nine clusters contain nine previously identified GBM related genes [34], including EGFR, MDM2, PDFGFA, MDM4, CDK4, MET (amplifications), CDKN2A, PTEN, and RB1 (deletions); however, MDM4 and RB1 lie slightly out of the detected region. These nine genes were also detected by GISTIC. In addition to these nine genes, aberrations of CDK6 and MYC have also been reported in multiple GBM studies [34]. However, using this GBM data set, aberrations of these two genes were detected neither by our method nor by the GISTIC method. The above results indicate that our method highly ranks most of the important GBM genes and reports only a small number of false positive positions--although there is a possibility that these false positive regions may include previously unreported GBM genes.

Table 1 shows the positions of clusters in chromosomes, ranked according to the cluster scores when *M *= 12. WIFA identified ten additional regions (other than the above nine genes). Two regions include known cancer related genes (MYCN [35] and IGFBPL1 [36]), though the remaining eight regions do not contain any cancer related genes; Additional File 2 contains the gene symbols for all clusters. In contrast, GISTIC identified 17 additional regions. In these regions, five known cancer related genes are included, one of which is MYCN. The remaining 12 regions do not contain any cancer related genes. Hence, both methods contain regions that may require further analysis, or they might just be falsely detected regions. Let us now revisit Figure 3 in order to illustrate the use of the HIGH transform to detect focal aberrations in GBM data [17]. Figure 3(a) presents the SNP array data of 154 patients at positions from 52,767 to 55,790 KB in chr7. In this region, DNA amplification seems apparent in multiple patients. Figure 3(b) then gives the HIGH analysis for the corresponding region. Note that the cluster from 54,145 to 55,790 KB ranks 2nd in Table 1 and contains EGFR.

**Table 1:** Clusters with focal aberrations in GBM.

Score	***P***_**cluster**_	Cytoband	Start (KB)	End (KB)	# of PA	Gene Symbol§
-363	0	9p21.3,p22.1	19,639	24,327	42	CDKN2A
266	0	7p11.2	54,145	55,790	25	EGFR
101	0	4q12	52,600	55,926	9	PDGFRA
66	0	1q32.1	200,858	202,110	5	MDM4†
60	0	12q15	67,074	68,482	6	MDM2
36	0.015	12q13.3,q14.1	55,820	57,257	4	CDK4
-35	0	13q14.2	46,386	47,510	3	RB1†
23	0.067	7q31.2	115,813	116,895	2	MET
-18	0	10q23.2,q23.31	88,974	89,943	3	PTEN
-16	0	19q13.2,q13.31	46,084	48,423	3	
-15	0	9p21.1	32,101	32,432	3	
8	0.001	17q22	47,672	49,744	2	
-7	0.034	1p33	50,351	50,689	2	
6	0	2p24.3	15,746	16,670	2	MYCN
-4	0	9p13.1	38,288	39,006	3	IGFBPL1
2	0.005	14q31.3	84,913	86,323	2	
-2	0	9p12	40,722	41,675	3	
-0.5	0	3p14.2	60,046	60,153	2	
-0.2	0.033	14q21.2,q21.3	42,887	43,214	2	

19 statistically significant clusters obtained from the HIGH analysis are shown when *M *= 12. 'Score' is the sum of  values for positions in the cluster. '*P*_cluster_' is the statistical significance of the cluster. Positive (negative) values represent that a cluster contains amplified (deleted) focal aberrations. Clusters are ordered by the absolute value of the score. Cytoband, the start and end positions of cluster regions, the number of patients with focal aberrations (# of PA), and GBM or cancer related genes included in the cluster are indicated.

§Gene annotations are based on hg18 human genome assembly.

†These genes are closely located to the focal aberrations. MDM4 is located at chr1:202,752-202,794 KB, and RB1 at chr13:47,775-47,954 KB.

We also applied the proposed method to a GBM data set obtained by Kotliarov *et al. *[20]. We used the same parameter values for *C*, *L*, *M*, and *d *as for the previous GBM data, since both are generated using the same SNP array platform. The HIGH analysis for this GBM data generates eight clusters; among these clusters, one focal deletion contains CDKN2A, and five focal amplifications contain MDM4, PDGFRA, EGFR, MDM2, and CDK4 (Additional File 3 and Additional File 4). Note that MET is not included in the focal aberrations because amplification occurs only in a single sample. The six GBM genes identified using this data are also found in the previous GBM data set. This result confirms that our method is able to detect focal aberrations that are consistent across different experiments.

### Broad and focal aberrations in lung cancer

We applied our method to the 371 lung cancer patients studied by Weir *et al. *[21]. Let us first explain how some of the parameters in our Methods section can be determined for a data set other than GBM data, by using this data set as an example. We recall that the parameters used for GBM data are *C *= 1.94, *L *= 9, *M *= 12, and *J_total _*= 17. Since the average distance between probes for GBM data set is 50 KB, the actual length of genomes we average for *y_LOW _*is approximately

and the actual length of genomes we consider as a focal aberration for *y_HIGH _*is approximately

For the lung data set, the average distance between probes is 13 K and *J_total _*:= ⌈log 2(*n_total_*)⌉ = 18, where *n_total _*is the number of total probes in the data set. Since we want the actual length of genomes that we average for *y_LOW _*and the actual length of genomes we consider as focal aberrations to be similar to the GBM case, we select *L *= 8 and *M *= 11. Then, the actual length of genomes we average over for *y_LOW _*is approximately

and the actual length of genomes that we consider as focal aberrations for *y_HIGH _*is approximately

With the value of *C *= 1.94, the number of nonzero values in *y_HIGH _*of all chromosomes in the GBM data set is about 10% of the number of nonzero values in the sample. The value of *C *that provides approximately the same percentage of nonzero values in *y_HIGH _*for the lung data set is *C *= 5.2.

Using the LOW analysis, we then attempt to detect broad aberrations in lung cancer. For this task, the *q*-value is first calculated for each probe. As shown in Additional File 5 (a) and 5 (b), with a threshold *q*-value of 0.01: chr1p, 3p, 4q, 5q, 6q, 8p, 9, 10q, 13p, 15, 16q, 18, 21p, and 22 are deleted; and chr1q, 2p, 5p, 6p, 7, 8q, 14p, 17q, and 20q are amplified in the size of the chromosome arm.

When Weir *et al. *[21] analyzed this lung cancer data set using the GISTIC approach, they identified five deleted focal regions and 17 amplified focal regions with statistical significance: in these regions, ten known oncogenes (MDM2, MYC, EGFR, CDK4, KRAS, CCNE1, ERBB2, CCND1, TERT, and ARNT), two known tumor suppressor genes (CDKN2A and PTEN), and six new candidate genes (MBIP, NKX2-1, VEGFA, PTPRD, PDE4D, and AUTS2) were found. We applied the WIFA approach to this same data set (*d *= 50 KB). When *M *= 11, 20 clusters spanning 28 MB are generated. Table 2 and Additional File 6 present a detailed description of these 20 clusters. The top-ranked cluster contains MBIP, a new candidate gene that is also ranked at the top in the GISTIC analysis. Among the 18 cancer related genes noted above, six known oncogenes (MDM2, EGFR, KRAS, CCNE1, ERBB2, and CCND1), one known tumor suppressor gene (CDKN2A), and three new candidate genes (MBIP, NKX2-1, and VEGFA) are included in the focal aberrations. In addition, SS18 and FGFR1--which were identified by Weir *et al.*, though they did not consider them statistically significant--are identified as having statistical significance in our study. Let us now look at the remaining clusters that do not contain cancer related genes reported by Weir *et al. *We investigated whether or not these remaining clusters contain other cancer related genes (Table 2). Two genes SMAD7 (chr18q21.1) and FGF10 (chr5p12) are located in focal aberration regions. It is known that SMAD7 functions as an intracellular antagonist of transforming growth factor beta (TGF-beta) signaling and is frequently unregulated in various cancers [37]. A recent study showed that a transgenic mouse model with SMAD7 disrupted TGF-beta signaling and increased lung carcinogenesis [38]. In our study, the values of  in chr18:43,953-45,322 KB, where SMAD7 is located, are positive in two patients. Indeed, the averages of the log2 intensity ratio of these two patients are higher than those of patients having zero values in  (Additional File 7(a)). This result implies the DNA amplification of SMAD7 in lung cancer. A member of the fibroblast growth factor FGF10 has also been reported in several cancer studies; multiple lines of evidences show that FGF10 plays important roles in various cancer types, including prostate and pancreatic cancers [39,40]. Previously, the mRNA expression of FGF10 in the fetal lung of mice was shown to disrupt lung morphogenesis [41], and the alternation of FGF pathways frequently occurs in lung cancer [42]. Our analysis reveals that eight lung cancer patients contain DNA amplification in the chr5:42,891-44,452 KB regions, including FGF10. As shown in Additional File 7(b), the average values of the log2 intensity ratios of samples having positive values in  are higher than those of samples having zero values in . This result suggests the possibility that the DNA copy numbers of FGF10 increase in lung cancer and might affect lung cancer development.

**Table 2:** Clusters with focal aberrations in lung cancer.

Score	***P***_**cluster**_	Cytoband	Start (KB)	End (KB)	# of PA	Gene Symbol§
118	0	14q13.2 q13.3	35,467	36,690	10	MBIP,NKX2-1
107	0	12p11.23 p12.1	24,055	26,685	4	KRAS
84	0	18q11.2 q12.1	20,300	23,537	4	SS18
62	0	7p11.2 p12.1	53,796	55,553	3	EGFR
54	0.006	18q21.1	43,971	45,322	2	SMAD7 ‡
51	0.064	12q15	67,983	69,669	4	MDM2 †
47	0.006	19q12	35,835	36,303	2	CCNE1 †
47	0	11q13.2 q13.3	68,164	69,500	3	CCND1
42	0.012	19q13.11 q13.12	37,716	41,040	5	
35	0	22q11.21	19,057	19,785	2	
33	0	17q12 q21.1	33,845	35,540	4	ERBB2
26	0.018	8p11.23 p12	38,417	39,171	2	FGFR1
24	0	10p11.21	37,594	38,717	2	
22	0	6p21.33	30,143	30,911	2	
15	0.023	6p22.1 p22.2	26,089	26,937	2	
14	0.007	5p12	42,982	44,452	2	FGF10 ‡
10	0.064	6p21.1	43,240	44,227	2	VEGFA
10	0.019	10q11.21	42,184	43,133	2	
9	0	9p21.3	24,546	25,375	2	
-3	0	9p21.3	21,181	22,194	2	CDKN2A

When *M *= 11, 20 statistically significant clusters are shown. These clusters contain known lung cancer genes or cancer related genes in other cancer types. All genes contained in the 20 clusters are described in Additional File 6. Columns are as described in Table 1.

§Gene annotations are based on hg18 human genome assembly.

†MDM2 is located at chr12:67,488-67,520 KB and CCNE1 at chr19:34,500 KB.

‡Cancer related genes identified using the proposed method, not GISTIC.

**Figure 5:**
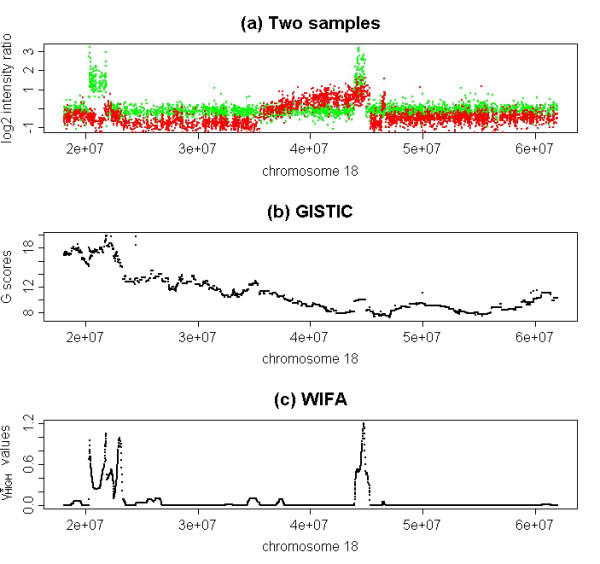
**Chr18:18-62 MB regions**. Both GISTIC and WIFA identify amplifications in chr18:20,300-23,559 KB, whereas amplifications in chr18:43,954-45,322 KB including SMAD7 are only identified by WIFA. (a) log2 ratios of two patients amplified in chr18:43,954-45,322 KB are shown. (b) G-scores in the same region by GISTIC are not large enough to be identified. (c)  by WIFA is significantly higher in the same region.

Let us further explain the difference between GISTIC and WIFA by looking at chr18:43,953-45,322 KB (where SMAD7 is located), which was identified only by WIFA. Figure 5(a) shows the log2 intensity ratios of two patients for part of chr18. Although both samples are commonly amplified in chr18:43,953-45,322 KB, the G-values obtained by GISTIC are not large enough to identify this region. The G-values are calculated by summing the log2 intensity ratios of the amplified samples; 40 samples are considered amplified in this region (log2 ratio>0.1). However, even though the original data from two patients (Figure 5(a)) were highly amplified and their differences with neighbors seemed significant, the G-values did not capture this significance. This is because the other 38 (amplified with less difference between neighboring values than the two) samples were also used to calculate the G-values, which resulted in the significance of the two samples being obscured (Figure 5(b)). In contrast, WIFA first calculates the differences with neighbors for each sample, then sums the differences. In WIFA, the  values are high for the two samples, but the  values are zero for the other samples since these other samples do not have any significant difference with their neighbors. Therefore, --the summation of  values from multiple samples--can reflect the significant differences with the neighbors of these two samples (Figure 5(c)). Note that both GISTIC and WIFA identify amplification in chr18:20,300-23,559 KB.

To validate our choice of *M*, we conducted experiments with various *M *values. For example, with *M *= 9, 37 clusters spanning 161 MB included 12 cancer related genes, as shown in Additional File 8(a). However, even though the *M *= 9 case identified two more genes compared to *M *= 11, it required the search of five times more genomic regions; refer to Additional File 8 for the results of the other *M *values. Our analysis of lung cancer confirms that WIFA is useful for identifying cancer related genes in focal aberrations across different cancer types.

### Comparison with other methods

We then compared our method with MCR and STAC. For implementation, we used the MCR from waviCGH [13](http://wavi.bioinfo.cnio.es/) and STAC from the authors' website (http://www.cbil.upenn.edu/STAC/). Note that the input files from both methods should have binary aberration calls of amplification, deletion, or no change; hence, GLAD [10], a segmentation method, was applied to single samples. The thresholds for amplification and deletion were then used to determine the aberration regions. In MCR, the fraction of samples in aberrant regions was used to determine the significant regions. In STAC, the *p*-value of the footprint was used as a measure of the significance of aberrant regions. For WIFA, the cluster score is used for this purpose.

We used a series of simulation data as the basis of our comparison, and generated the simulation data in two steps. First, ten different underlying true data were generated using Multiple Sample Analysis [11](http://www.cbil.upenn.edu/MSA/) software. For each true data, the length of a genome, in terms of number of markers, was 4,500; in addition, the number of samples was 50; the number of markers in the underlying concordant aberrations was 30; and the numbers of samples in concordant aberrations varied from 50% to 70%. In ten true data, the numbers of concordant aberrant regions varied from five to seven, and there were one or two nonconcordant regions. Second, the background aberrations were generated using a normal distribution. Because the maximum (in absolute) values of the markers for each sample were different, we set the standard deviation of the normal distribution to be the multiplication of a fixed number, which we refer to as the noise level, and the maximum value of the true data.

**Figure 6:**
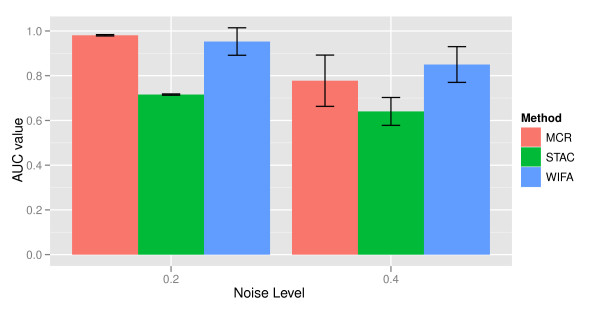
**Comparison of STAC, MCR, and WIFA in the simulation data**. Averages of AUC values of ten underlying true data in three methods are drawn for noise levels of 0.2 and 0.4. The standard deviation of the AUC values for ten underlying true data is drawn as error bars.

We investigated noise levels of 0.2 and 0.4. Performances of three methods are measured based on values for the area under a curve (AUC) for the sensitivity and false positive rate. For both noise levels, WIFA shows very good performance in identifying concordant regions in the simulation data, as shown in Figure 6. Note that MCR performs slightly better than WIFA when the noise level is 0.2, but WIFA is superior when the noise level is 0.4. Considering that a lower noise level generally results in easier noise removal, we can conclude that WIFA is the most useful in identifying concordant regions in the simulation data. During these simulations, the parameter values of *C*, *L*, and *M *in WIFA were determined in the same manner as for lung cancer. In MCR and STAC, 0.1 is used as the threshold value for identifying amplifications and deletions in a single sample, after application of the GLAD segmentation method.

**Figure 7:**
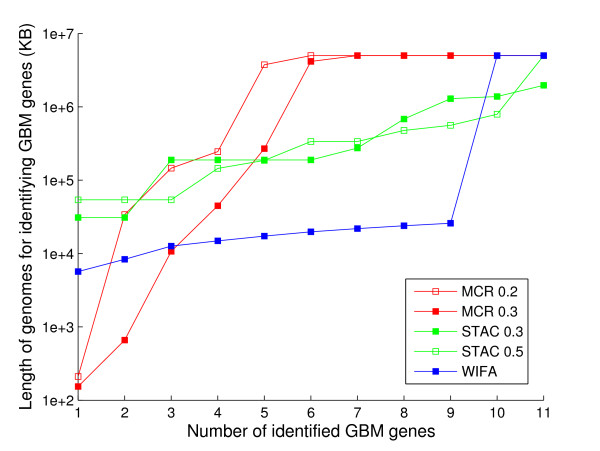
**Comparison of STAC, MCR, and WIFA in GBM data**. The *y*-axis is the length of the genome regions sorted based on the significance of regions, and the *x*-axis is the number of GBM genes contained in the corresponding genomes in the *y*-axis. When the genome length is larger than 6,000,000 KB, it is shown exactly as 6,000,000 KB for clarity. Because methods with high performance require a smaller length of genomes in their search for known GBM related genes, a method closer to *x*-axis is considered to outperform other methods.

We next compared WIFA with MCR and STAC using real GBM data sets. As a measure of performance, we used the length of genomes to identify known GBM genes. In Figure 7, the *y*-axis represents the length of the genome regions sorted based on the region significance, and the *x*-axis represents the number of GBM genes contained in the corresponding genomes in the *y*-axis. Because methods with high performance require a smaller length of genomes in their search for known GBM related genes, methods closer to the *x*-axis generally outperform other methods. Here, on average, WIFA typically shows the best performance, as nine genes can be identified around 10,000 KB. In MCR and STAC, five different thresholds of 0.1, 0.2, 0.3, 0.4, and 0.5 are used to determine the amplification and deletion. In both methods, the threshold value of 0.3 shows the best performance. Figure 7 includes the graphs of the 0.3 threshold, along with one other threshold for comparison. In MCR, only three or four genes are identified within 10,000 KB; more than 100,000 KB is required to identify the remaining genes. In STAC, more than 10,000 KB is required to identify most of the genes.

## Discussion and Conclusions

Our work is based on a wavelet analysis. The wavelet analysis has been used in other papers to analyze array CGH data (cf. [9], [43]); for example, in [9], it is shown to perform well compared to approaches such as CBS, a change-point method [8], and HMM [44]. Compared to other wavelet-based approaches used to analyze array CGH data, the main differences in WIFA include: (i) a new parameter *M *is introduced, which is used to identify focal genomic aberrations more effectively; and (ii) a new method that integrates multiple samples, as a post-processing step in the wavelet analysis, is suggested in order to identify cancer-related genes from a data set having multiple samples. As a result, we were able to detect cancer related genes with high rate of accuracy in both GBM and lung data sets.

CNPs are another type of DNA variation that are abundant in the normal population, and are usually observed in kilobase or megabase DNA deletions or duplications. When a HIGH analysis was applied to SNP microarrays, deletions of a single SNP probe were frequently observed. When these were compared to the positions of known CNPs [22], many regions were found to overlap (data not shown); these single SNP probes were removed from our analysis since the relevance of CNPs to cancer requires further study. However, if CNPs are the main subject of analysis, it is possible that a new method based on our HIGH analysis could be developed to achieve this task. As a promising example, a single deletion of the SNP probe from 13 patients was observed at the 55,205,890 base position of chr11 when the GBM data set was used [17]. Olfactory receptor (OR) genes such as OR4C11, OR4P4, OR4S2, and OR4C6 are located at this position, and it was previously shown that the OR genomic location is frequently affected by CNPs [45]. This observation suggests that our wavelet analysis has the potential to be broadly applied to detect various kinds of focal aberrations.

## Authors' contributions

YH developed and implemented the proposed method, and wrote the manuscript. HL initiated the project, developed and implemented the proposed method, and wrote the manuscript. All authors read and approved the final manuscript.

## Supplementary Material

Additional file 1 **Broad aberrations in GBM data **[17]. Broad aberrations of GBM data [17] are shown with a *q*-value threshold of 0.01: (a) deletions are shown in chr6q, 9p, 10, 13, 14, and 22, and (b) amplifications are shown in chr7, 8q, 17q, 19p, and 20. Click here for file

Additional file 2**Clusters with focal aberrations in GBM data set **[17]. 19 clusters using HIGH analysis are shown when *M *= 12. 'Score' is the sum of  values for positions in the cluster. Positive (or negative) value represents that a cluster contains amplified (deleted) focal aberrations. Clusters are ordered by the score. 'Pcluster' is a statistical significance of the cluster. Chromosome, cytoband, start and end positions of cluster regions, the number of patients with focal aberrations, and genes in the focal aberrations are shown. Gene annotations are based on hg18 human genome assembly.Click here for file

Additional file 3 **Focal aberrations in GBM data **[20]. Focal aberrations of GBM data [20] are shown with *M *= 12. Deletions (amplifications) are indicated in blue (red). Focal deletions contain CDKN2A, and focal amplifications contain MDM4, PDGFRA, EGFR, MDM2, and CDK4. Click here for file

Additional file 4**Clusters with focal aberrations in GBM data set **[20]. 8 clusters using HIGH analysis are shown when *M *= 12. 'Score' is the sum of  values for positions in the cluster. Positive (or negative) values indicate that a cluster contains amplified (deleted) focal aberrations. Clusters are ordered by score. 'Pcluster' is the statistical significance of the cluster. Chromosome, cytoband, start and end positions of cluster regions, the number of patients with focal aberrations, and genes in the focal aberrations are shown. Gene annotations are based on hg18 human genome assembly.Click here for file

Additional file 5 **Broad aberrations in lung cancer data **[21]. Broad aberrations of lung cancer data are shown for a *q*-value threshold of 0.01. (a) Deletions are shown in chr1p, 3p, 4q, 5q, 6q, 8p, 9, 10q, 13p, 15, 16q, 18, 21p, and 22. (b) Amplifications are shown in chr1q, 2p, 5p, 6p, 7, 8q, 14p, 17q, and 20q. Click here for file

Additional file 6**Clusters with focal aberrations in lung data set **[21]. 20 clusters from the HIGH analysis are shown when *M *= 11. 'Score' is the sum of  values for positions in the cluster. Positive (or negative) values indicate that a cluster contains amplified (deleted) focal aberrations. Clusters are ordered by score. 'Pcluster' is the statistical significance of the cluster. Chromosome, cytoband, start and end positions of cluster regions, the number of patients with focal aberrations, and genes in the focal aberrations are shown. Gene annotations are based on hg18 human genome assembly.Click here for file

Additional file 7**log2 intensity ratio of patients in the regions including SMAD7 and FGF10**. (a) In the region chr18:43,954-45,322 KB (133 probes), where SMAD7 is located, two patients have positive  values. For each probe, the average values of intensities of the two groups of patients are plotted: 'Group1' contains patients having positive values in  and 'Group2' contains patients having zero values in . (b) In the region chr5:42,891-44,452KB (77 probes), where FDF10 is located, eight patients have positive values in . In both cases, it is clearly shown that the log2 intensity ratio is higher in patients having positive values in  than samples having a zero value in .Click here for file

Additional file 8 **Focal aberrations in lung cancer data for several *M *values**. Focal aberrations of lung cancer data [21] are shown. *M *values used range from 9 to 12. (a)-(d) As described in Figure 4(a)-(g) in the main text. Click here for file
