# Supplementary figures and images for: Wavelet-based identification of DNA focal genomic aberrations from single nucleotide polymorphism arrays
Source: BMC Bioinformatics. 2011 May 11;12:146. doi: 10.1186/1471-2105-12-146 (PMC3114745; doi:10.1186/1471-2105-12-146)

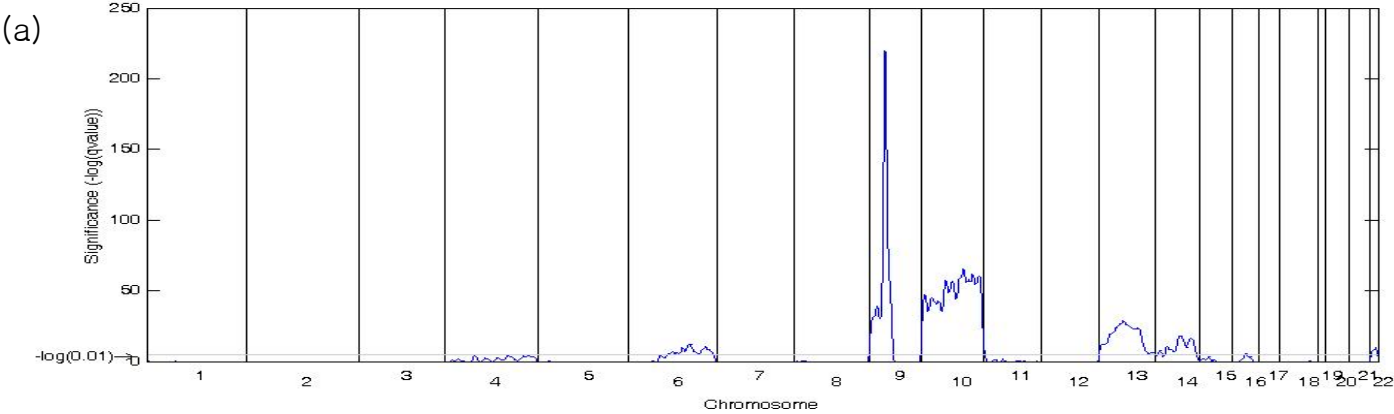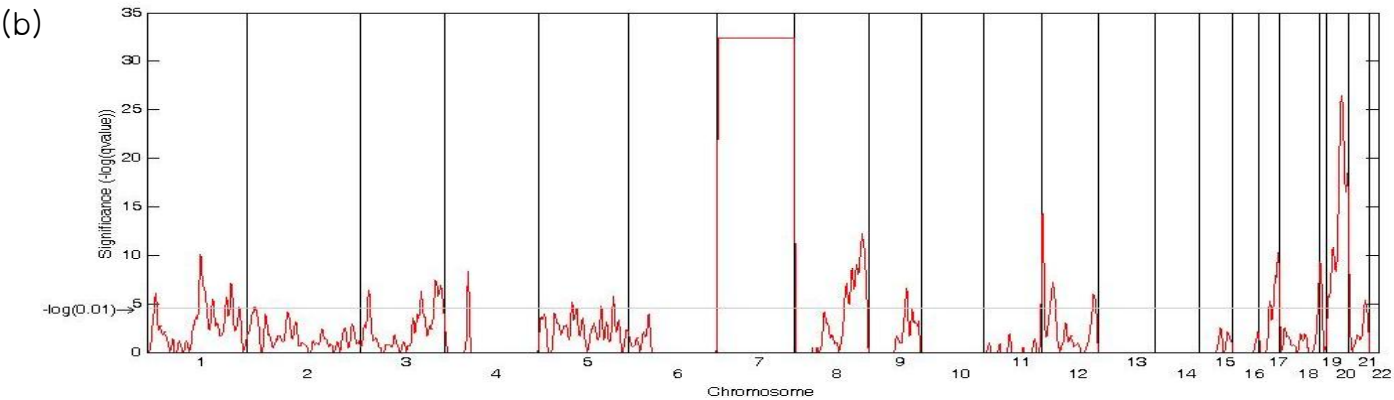

Supplement: Additional file 1 — Broad aberrations in GBM data [17]. Broad aberrations of GBM data [17] are shown with a q-value threshold of 0.01: (a) deletions are shown in chr6q, 9p, 10, 13, 14, and 22, and (b) amplifications are shown in chr7, 8q, 17q, 19p, and 20. [file 1471-2105-12-146-S1.PDF]

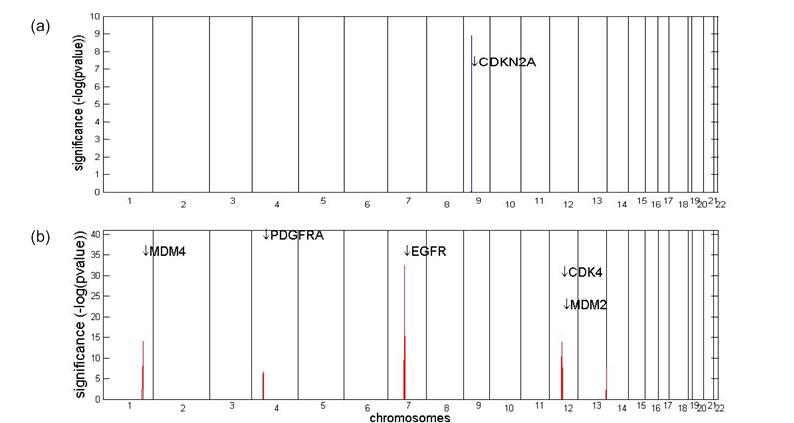

Supplement: Additional file 3 — Focal aberrations in GBM data [20]. Focal aberrations of GBM data [20] are shown with M = 12. Deletions (amplifications) are indicated in blue (red). Focal deletions contain CDKN2A, and focal amplifications contain MDM4, PDGFRA, EGFR, MDM2, and CDK4. [file 1471-2105-12-146-S3.JPEG]

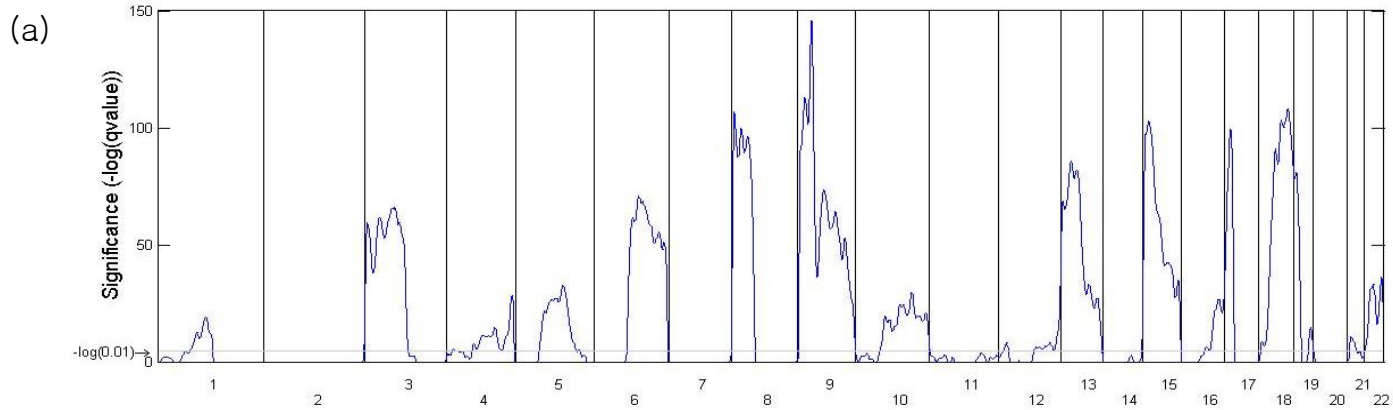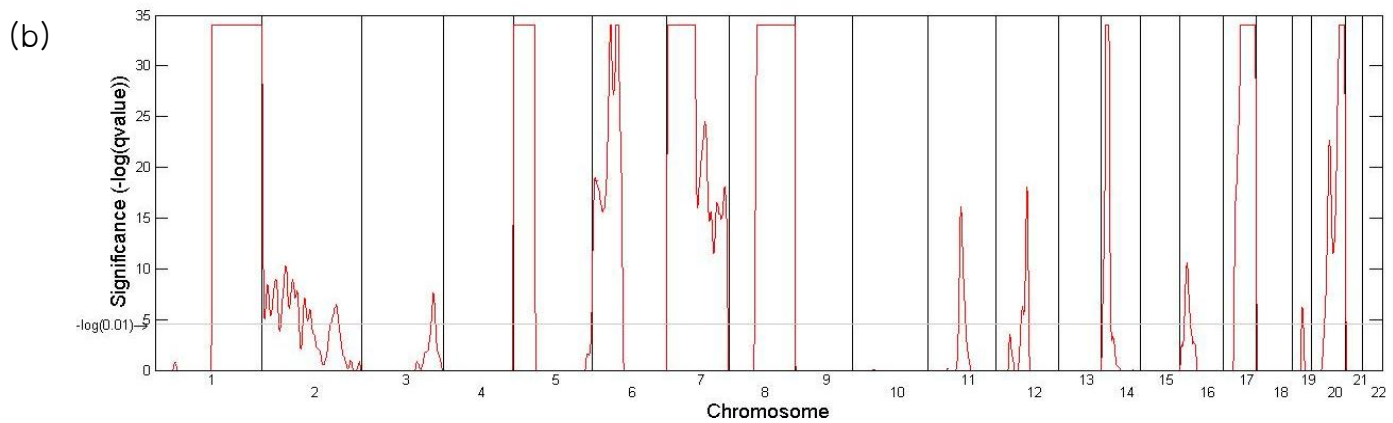

Supplement: Additional file 5 — Broad aberrations in lung cancer data [21]. Broad aberrations of lung cancer data are shown for a q-value threshold of 0.01. (a) Deletions are shown in chr1p, 3p, 4q, 5q, 6q, 8p, 9, 10q, 13p, 15, 16q, 18, 21p, and 22. (b) Amplifications are shown in chr1q, 2p, 5p, 6p, 7, 8q, 14p, 17q, and 20q. [file 1471-2105-12-146-S5.PDF]

(a)

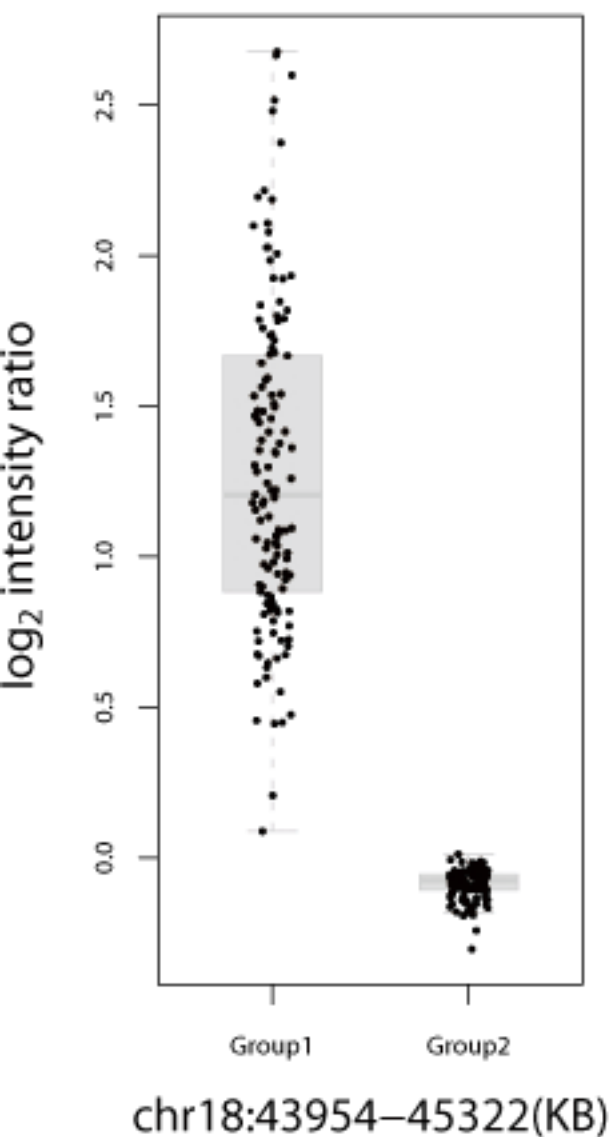

(b)

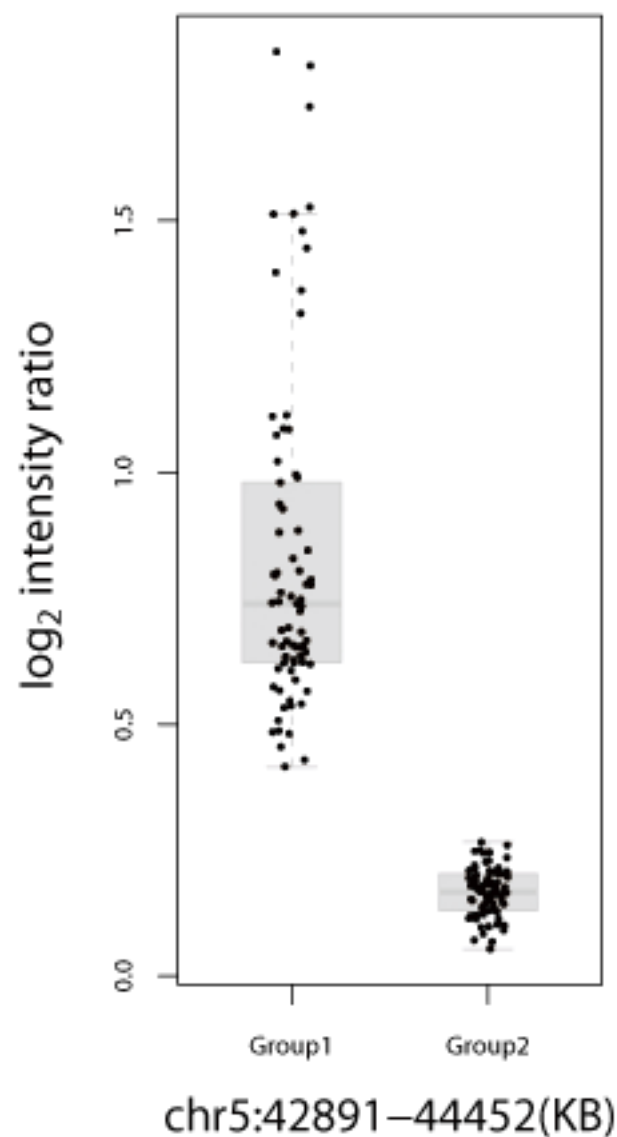

Supplement: Additional file 7 — log2 intensity ratio of patients in the regions including SMAD7 and FGF10. (a) In the region chr18:43,954-45,322 KB (133 probes), where SMAD7 is located, two patients have positive values. For each probe, the average values of intensities of the two groups of patients are plotted: 'Group1' contains patients having positive values in and 'Group2' contains patients having zero values in . (b) In the region chr5:42,891-44,452KB (77 probes), where FDF10 is located, eight patients have positive values in . In both cases, it is clearly shown that the log2 intensity ratio is higher in patients having positive values in than samples having a zero value in . [file 1471-2105-12-146-S7.PDF]
