# Supplementary material for: Wavelet-based identification of DNA focal genomic aberrations from single nucleotide polymorphism arrays
Source: BMC Bioinformatics. 2011 May 11;12:146. doi: 10.1186/1471-2105-12-146 (PMC3114745; doi:10.1186/1471-2105-12-146)
Supplement: Additional file 8 — Focal aberrations in lung cancer data for several M values. Focal aberrations of lung cancer data [21] are shown. M values used range from 9 to 12. (a)-(d) As described in Figure 4(a)-(g) in the main text. [file 1471-2105-12-146-S8.PDF]

(a) M = 9

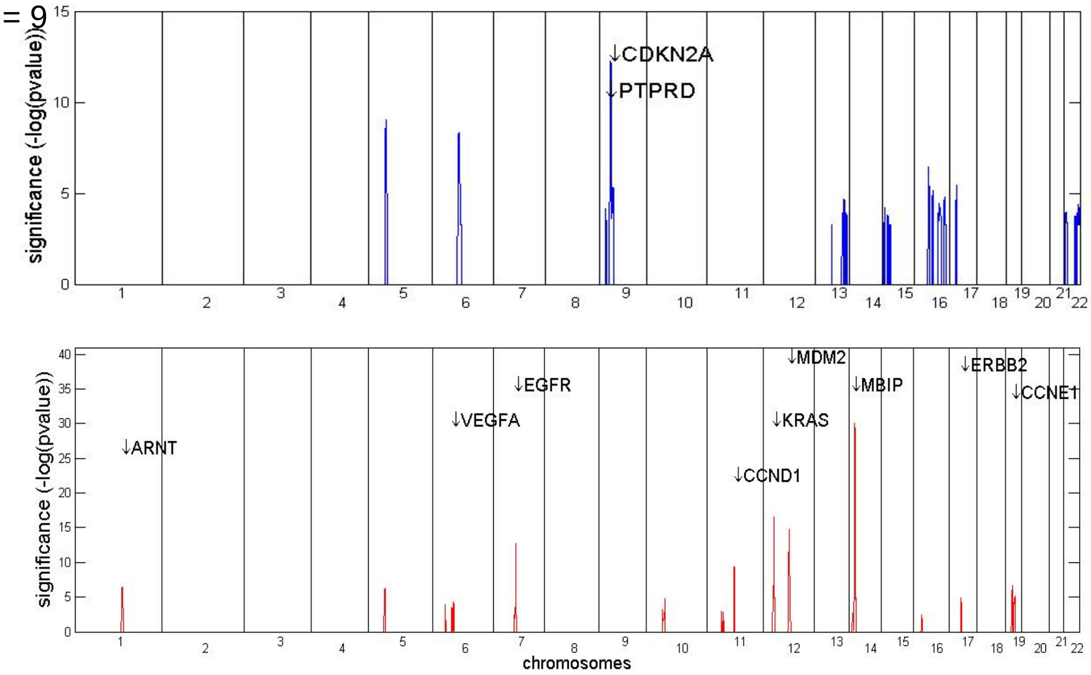

Rank	Gene
1	MDM2
2	MBIP,NKX2-1
3	KRAS
4	CCNE1
5	CDKN2A
6	ARNT
7	EGFR
8	
9	CCND1
10-14	...
15	VEGFA
16-23	...
24	ERBB2
25-35	...
36	PTPRD
37	

161MB

12/37

(b) M=11

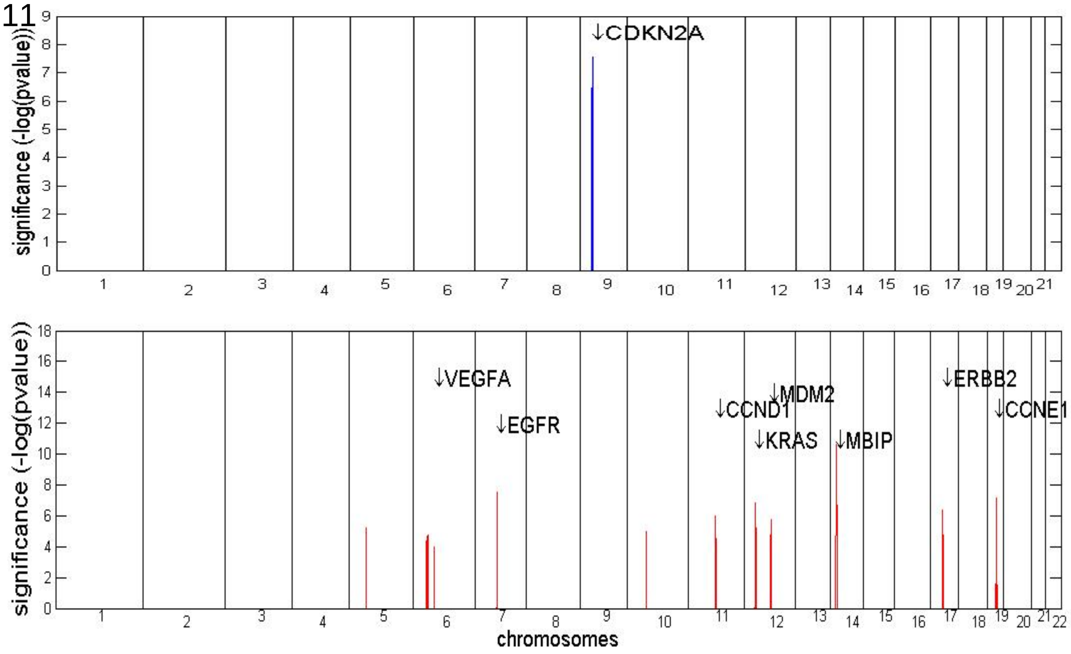

Rank	Gene
1	MBIP,NKX2-1
2	KRAS
3	
4	EGFR
5	
6	MDM2
7	CCNE1
8	CCND1
9-10	...
11	ERBB2
12-16	...
17	VEGFA
18-19	...
20	CDKN2A

28MB

10/20

(c) M=10

Rank	Gene
1	MBIP,NKX2-1
2	KRAS
3	MDM2
4	CCNE1
5	EGFR
6	
7	FGFR1
8	CCND1
9	
10	CDKN2A
11	VEGFA
12	
13	ERBB2
14-25	...

61MB

11/25

(d) M=12

Rank	Gene
1	EGFR
2	MBIP,NKX2-1
3	ERBB2
4-5	...

8MB

4/5
